# Environmental contamination and cleaning practices in long-term care: a transdisciplinary mixed-methods study

**DOI:** 10.1017/ash.2026.10324

**Published:** 2026-04-07

**Authors:** Morgan Jane Katz, Alejandra B. Salinas, Heather Stoltzfus, Yea-Jen Hsu, Kellogg Schwab, Natalie Exum, Abigail Vorsteg, Matiza Florena Sacotingo, Caroline Coulter, Sara Cosgrove, Clare Rock

**Affiliations:** Johns Hopkins University School of Medicine, Baltimore, MD, USA

## Abstract

**Objective:**

To identify system-level barriers to effective environmental cleaning and disinfection in long-term care (LTC) facilities.

**Design:**

Transdisciplinary, mixed-methods approach using human factors engineering and environmental sampling to assess environmental cleaning practices and associated multi-drug resistant organism (MDRO) burden in LTC patient rooms and common areas.

**Setting:**

Two high-acuity skilled nursing facilities in Maryland.

**Methods:**

Environmental sampling of high-touch surfaces (HTSs) was performed in resident rooms, communal areas, and nursing stations. Samples were analyzed for MDROs and overall bacterial burden. System-level barriers were assessed through observations of cleaning and focus groups with environmental services (EVS) staff and supervisors, guided by the Systems Engineering Initiative for Patient Safety (SEIPS) model.

**Results:**

Of 123 composite samples of environmental surfaces, 76% of patient room samples were culture positive for 1+ MDRO, with MRSA the most prevalent organism. Quantitative bacterial burden was higher from Composite 1 (the area in the room closest to the resident) than any other composite in the resident room (median of 27.17 CFU/mL/cm^2^ vs 4.55 (near door) and 1.22 (bathroom)). Observations indicated significant deviations from cleaning protocols, with only 43% of HTSs cleaned per facility policy. Focus group discussions highlighted systemic challenges, including inconsistent training, frequent interruptions, and poor communication among staff.

**Conclusions:**

The findings underscore the burden of MDRO contamination in LTC settings and critical barriers to effective environmental cleaning. Addressing these issues through standardized training with feedback mechanisms, enhanced communication, and leadership engagement is essential for improving infection prevention efforts and resident safety in LTC facilities.

## Introduction

Long-term care (LTC) facilities are known reservoirs for the cultivation and transmission of multidrug-resistant organisms (MDROs) across the healthcare spectrum.^1^ Environmental cleaning, a cornerstone of infection prevention, is particularly challenging in LTC due to unique operational, resident, and environmental factors. Residents often reside in LTC for extended periods and regularly interact in communal areas–such as dining rooms, activity spaces, and therapy gyms–leading to the contamination of shared spaces.

While environmental cleaning and disinfection are essential in the LTC setting, implementation is often inconsistent.^2^ Preliminary data suggest that LTC environments have a higher burden of MDRO contamination than acute care facilities.^3–5^ A study of 28 California LTC facilities found that MDROs were recovered from 74% of resident rooms and 94% of common areas.^3^ Despite growing recognition of the need for effective environmental cleaning and disinfection in this high-risk setting, few studies have explored the system-level barriers that hinder consistent cleaning practices in LTC.^2,6–7^

We applied a novel transdisciplinary, mixed-methods approach to evaluate the extent of MDRO contamination and system-level barriers to cleaning/disinfection in two Maryland LTC facilities. Our team integrated expertise from various disciplines including human factors engineering, infection prevention, and environmental microbiology. We conducted environmental sampling of high-touch surfaces in resident rooms, common areas, and nursing stations and evaluated for the presence of MDROs as well as overall bacterial burden. Specific barriers to environmental cleaning practices were assessed via direct observations of the cleaning process and focus group discussions with environmental services (EVS) staff and supervisors.

## Methods

### Study population and setting

Two high-acuity skilled nursing facilities in Maryland, each privately owned by different corporate groups with distinct organizational structures, policies, and procedures. Both facilities provided complex wound care, off-site hemodialysis, and tracheostomy care; facilities were selected to evaluate different corporate structures and based on their ability to provide care to high-acuity patients likely to be colonized with MDROs.

Additional details about each facility’s structure, EVS policies, and organization are included in Supplementary Table 1.

This study was acknowledged by the Johns Hopkins University institutional review board as Non-Human Subjects research.

#### Environmental sampling

**Figure 1.**
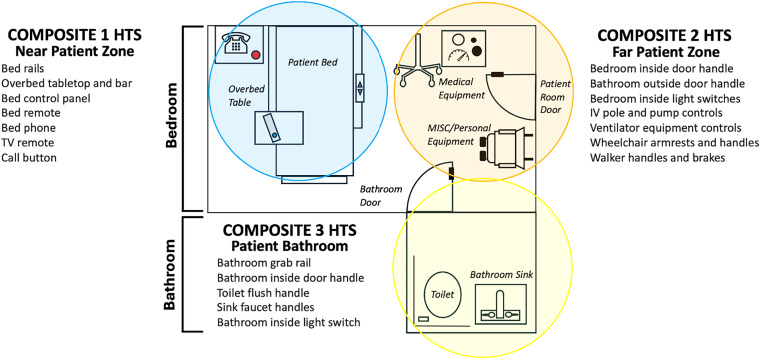
Visual representation of composite sampling of resident room and bathroom. HTS, high-touch surfaces; TV, television; MISC, miscellaneous; IV, intravenous.

We prospectively sampled high-touch surfaces (HTSs) from three areas in each facility: (1) resident rooms and bathrooms, (2) nurses’ stations, and (3) communal gyms. Resident room samples were further divided into three zones which we assigned Composites 1–3. (Figure 1) Composite 1 was used to sample HTSs directly around the resident (eg, bed rails, tray tables). Composite 2 sampled HTSs farther from the resident within the room (eg, light switches, medical equipment, doorknobs). Composite 3 sampled HTSs in the bathroom. Polyurethane stick sponges with neutralizer buffer (Hygiena™) were used for surface sampling, with up to 5 HTSs sampled using each side of the sponge, aiming for a total surface area between 175 and 2,500 cm^2^.

#### Laboratory processing

Sponges were homogenized in 45 mL PBS + 0.01% Tween 80 (PBS/T) using a Stomacher 400, centrifuged 3,300×g for 15 min, the supernatant discarded, and the pellet resuspended in 2 mL PBS/T for culture dilution on TSA + 5% sheep blood (TSA-SB) and McConkey plates, incubated at 35°C overnight, and colonies enumerated. To evaluate for methicillin-resistant *Staphylococcus aureus* (MRSA), and vancomycin-resistant enterococcus (VRE), we picked up to 10 colonies growing on TSA-SB and sub-cultured these to selective chromogenic agar plates (BBL Chromagar and Spectre VRE) and incubated overnight at 35°C. For extended-spectrum beta-lactamase-producing Enterobacterales (ESBL-E) bacteria and Carbapenemase-producing Enterobacterales (CPE), we picked up to 10 colonies growing on McConkey agar and sub-cultured onto HardyCHROM™ ESBL, and ChromID® Carba, chromogenic agar. For each composite, we calculated the density of bacterial CFUs per cm^2^ by dividing the number of CFUs from all sponges in that composite, by the surface area sampled. We conducted Wilcoxon rank sum tests to examine differences in bacterial density across composites and unit types. We then calculated the frequency of MDRO recovery overall, by LTCF, and the MDRO type. Each HTS was categorized according to the frequency of MDRO recovery: 0%–10%, 10%–20%, or >20%.

#### Environmental cleaning observations and focus groups

The Systems Engineering Initiative for Patient Safety (SEIPS)^8^ was used as the conceptual framework guiding our observations and qualitative focus groups (Figure 3). SEIPS is a human factors engineering model that focuses on how the 5 components of a work system–people, tasks, tools and technology, physical environment, and organization, and has been applied to environmental cleaning to influence work processes and outcomes.^8^

### Observations with contextual inquiry

Observations with contextual inquiry of environmental services (EVS) staff during daily and terminal cleans of resident rooms and common areas were conducted to identify gaps between “work-as-imagined” (policy and protocols) and “work-as-done” (real-world practices).

Observations were recorded on an Ipad using standardized observation forms to document various factors including cleaning duration, room characteristics (eg, number of people in room, presence of resident belongings), percentage of surfaces cleaned, products used, practice characteristics (eg, cleaning path, interruptions, hand hygiene, and personal protective equipment (PPE) compliance), and other deviations from facility policy.

Observers followed EVS associates throughout cleaning and conducted structured inquiries to elicit insights into decision making. Example inquiries included: “What might prevent you from entering a resident’s room to clean? If you are interrupted during cleaning, how do you remember to return to finish cleaning?”

Standardized observation guides are available in Supplementary Table 2.

### Focus groups

One-hour focus groups of 6–10 EVS staff or supervisors were conducted at each facility (Participant roles and demographics in Supplementary Table 3). Focus groups were informed by previous observations and based on the SEIPS model (Supplementary Figure 1). Discussions were audio-recorded, transcribed, and anonymized.

Four researchers (MK, HS, AS, AG) independently reviewed the focus group transcripts and assigned codes to segments of text. Codes were used to form the initial sub-themes and through the process of constant comparison these were grouped into higher-order themes to develop a thematic framework. The frameworks were combined, and any discrepancies were resolved through discussion.

## Results

### Quantitative

We collected 123 total composite samples of high-touch surfaces (HTS) (Table 1). Overall, 76% of samples from resident rooms were culture positive for at least one MDRO, and 15% grew two or more MDROs. Compared with resident rooms, common areas were less likely to be positive for any MDROs (nursing station 54%, gym, 43%, (*P* < .05). Ventilator and skilled units were more likely to have recovery of multiple MDROs than LTC units (15% in ventilator and skilled vs 4% in LTC). Facility 1 had a higher likelihood of recovering any MDRO (72% vs 53%) or 2 or more MDROs from all composites than Facility 2 (14% vs 5%).

**Table 1.** Percent MDRO positivity and quantitative bacterial burden by composite, facility, and room type

Area/composite	n	% of zones			% of zones				Quantitative bacterial burden (CFU/mL/cm^2^)^b^	
		Any MDRO^a^	1 MDRO	2+ MDROs	MRSA	ESBL	VRE	CPE	Median	Interquartile range
**All**	**123**	63	54	10	59	11	4	4	2.23	0.74–10.12
**Patient room–Composite 1**	23	78	65	13	65	13	13	4	27.17	8.04–68.14
**Patient room–Composite 2**	23	70	48	22	70	22	9	9	4.55	1.54–21.43
**Patient room–Composite 3**	21	81	71	10	71	19	0	0	1.22	0.29–2.72
**Nurse station**	28	54	50	4	50	4	0	4	1.40	0.57–2.23
**Gym**	28	43	39	4	43	4	0	4	1.23	0.13–4.49
**Facility 1**	**65**	72	58	14	72	12	5	5	1.41	0.46–9.41
**Patient room–Composite 1**	12	92	75	17	92	8	8	0	28.79	3.37–58.61
**Patient room–Composite 2**	12	83	50	33	83	33	17	17	6.98	2.34–36.43
**Patient room–Composite 3**	11	100	82	18	100	18	0	0	0.61	0.18–1.99
**Nurse station**	15	73	73	0	73	0	0	0	0.79	0.46–1.54
**Gym**	15	27	20	7	27	7	0	7	0.37	0.09–10.12
**Facility 2**	**58**	53	48	5	43	10	3	3	3.01	1.32–10.56
**Patient room–Composite 1**	11	64	55	9	36	18	18	9	26.88	14.38–80.49
**Patient room–Composite 2**	11	55	45	9	55	9	0	0	3.37	1.44–13.07
**Patient room–Composite 3**	10	60	60	0	40	20	0	0	2.31	0.44–8.15
**Nurse station**	13	31	23	8	23	8	0	8	1.91	1.32–2.53
**Gym**	13	62	62	0	62	0	0	0	2.89	1.06–3.54
**Unit type**										
**LTC**	28	75	71	4	68	7	0	4	2.60	1.11–27.96
**Rehab/skilled**	26	58	42	15	54	15	4	8	1.90	0.76–9.33
**Vent/ICU**	41	73	59	15	66	17	10	2	2.53	1.22–7.64
**Gym**	28	43	39	4	43	4	0	4	1.23	0.13–4.49

MDRO, Multidrug-resistant organisms; MRSA, methicillin-resistant staphylococcus aureus; ESBL, Extended-spectrum beta-lactamase-producing (ESBL) bacteria; VRE, Vancomycin-resistant enterococcus; CPE, Carbapenemase-producing Enterobacterales; PR, Patient room; LTC, Long-term care facility; ICU, Intensive care unit.

a Tested using chi-square tests or Fisher’s exact tests, the MDRO status was statistically different across composites/areas in Facility 1 (*P* < .05), but not in Facility 2. The MDRO status was also statistically different across unit types (*P* < .05). Specifically, gym areas were less likely to have any MDROs compared to LTC and Vent/ICU areas.

b Examined by Wilcoxon rank sum tests using data from both facilities, composites 1 and 2 in patient rooms had a significantly higher quantitative bacterial burden than other areas, including patient room composite 3, nurse stations, and gym areas (*P* < .05). Additionally, areas in the LTC unit had a significantly higher quantitative bacterial burden than gym areas (*P* < .05). Other differences were not statistically significant.

MRSA was the most prevalent organism across all composites in both facilities, with 59% of samples testing positive; the majority of these were from patient rooms (69% vs 50% in nursing stations and 43% in gyms). ESBL was positive in 18% of patient rooms, 4% of nursing station and gym composites. VRE was positive in 10% of patient rooms and not recovered in nursing stations or gyms. CPE was recovered in 4% of all surfaces.

Quantitative bacterial burden was higher from Composite 1 (the area in the room closest to the resident) than any other composite in the resident room or common area (Median of 27.2 CFU/ml/cm^2^ vs 4.6 (C2) and 1.2 (C3)) (*P* < .05 comparing C1 to all other composites). Facility 2 had a higher overall bacterial burden (3.0 CFU/ml/cm^2^) from sampled surfaces than facility 1 (1.4), despite having lower MDRO rates.

### Qualitative

We observed 27 daily and 4 terminal cleaning processes of resident rooms. Daily cleans took an average of 27 minutes while terminal cleans took an average of 90 minutes. If the resident or other health workers were present during cleaning, the average time taken by EVS to complete the clean increased to 33 minutes. EVS staff wore gloves 89% of the time (32/36 observations), and hand hygiene was performed upon room entry 40% of the time.

**Figure 2.**
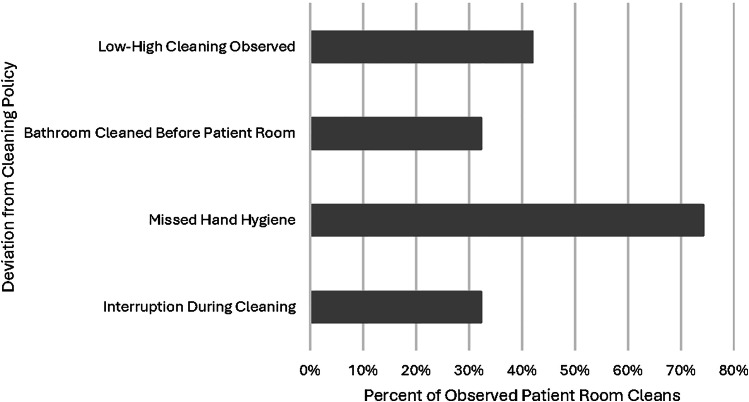
Observed deviations from advised facility cleaning policies (n = 31 observations).

43% (465/1,085) of HTSs were cleaned across all observations (Table 2). Deviations from facility policies were common. Observed deviations included not following a high then low surface order of cleaning (41%), incorrect sequence of bathroom cleaning (32%), missed hand hygiene (74%), and interruption of the cleaning process (32%) (Figure 2).

**Table 2.** Descriptive characteristics of patient room observations (n = 31)

Descriptive characteristics	n (%)
**Facility**	
1	19 (61)
2	12 (39)
**Unit type**	
LTC	13 (42)
Rehab/skilled	11 (35)
Vent /ICU	7 (23)
**Transmission-based precautions signage present**	
Yes	10 (32)
No	21 (68)
**If transmission-based precautions signage was present (n = 10), was appropriate PPE worn?**	
Yes	4 (40)
No	6 (60)
**Presence of resident/visitors**	
Neither	4 (13)
Resident only	25 (81)
Visitor only	1 (3)
Resident & visitor	1 (3)
**Estimated coverage of resident belongings on horizontal room surfaces**	
<25% of surfaces covered	8 (26)
25%–50% of surfaces covered	11 (39)
>50% of surfaces covered	12 (35)
**Type of room clean**	
Daily	27 (87)
Terminal	4 (13)
**Was hand hygiene performed upon room entry?**	
Yes	12 (39)
No	19 (61)
**Was hand hygiene performed upon room exit?**	
Yes	18 (58)
No	13 (42)
**Average number of HTS cleaned (n = 35 HTS)**	
LTC (13 rooms)	12/35 (34)
Rehab/skilled (11 rooms)	20/35 (57)
Vent/ICU (7 rooms)	12/35 (34)
Overall (31 rooms)	15/35 (43)

LTC, long-term care; ICU, intensive care unit; PPE, personal protective equipment; HTS, high-touch surfaces.

### Focus *groups*

Focus group discussions highlighted several challenges to effective environmental cleaning. Staff described inconsistent cleaning practices due to informal, peer-led training and the absence of standardized protocols. Frequent interruptions from residents, healthcare staff, and visitors were cited as a common challenge, often disrupting workflow, and leading to incomplete cleaning. Poor communication between EVS and nursing staff, especially around room access, patient movement, and the handling of bodily fluids, contributed to role confusion and frustration among staff members. One CNA said, “*The nurses. They disrespect. Like you could clean a room and they will go in there and change a patient’s dressing and throw it on the floor.*” Staff also reported short staffing and late notifications about room turnovers as a major barrier to thorough cleaning. One supervisor said: “*we can’t, you know, do terminal cleans, if staffing is down.*” Staff also reported difficulty with communication around when terminal cleans are occurring. When asked who was responsible for communication about scheduling terminal cleans, staff said “*No, I think we all just really don’t know*”; others chimed in–“*they don’t tell us.”*

Additional challenges included environmental clutter and the need to defer to resident requests, which limited access to high-touch surfaces. One staff member commented: *“Sometimes they’ll move their stuff for you so you can wipe the table, but if there’s like a lot of food, [you’re] not really supposed to touch their food and stuff, so unless they move it, you’re kind of– (Staff, Facility 1).”*

**Figure 3.**
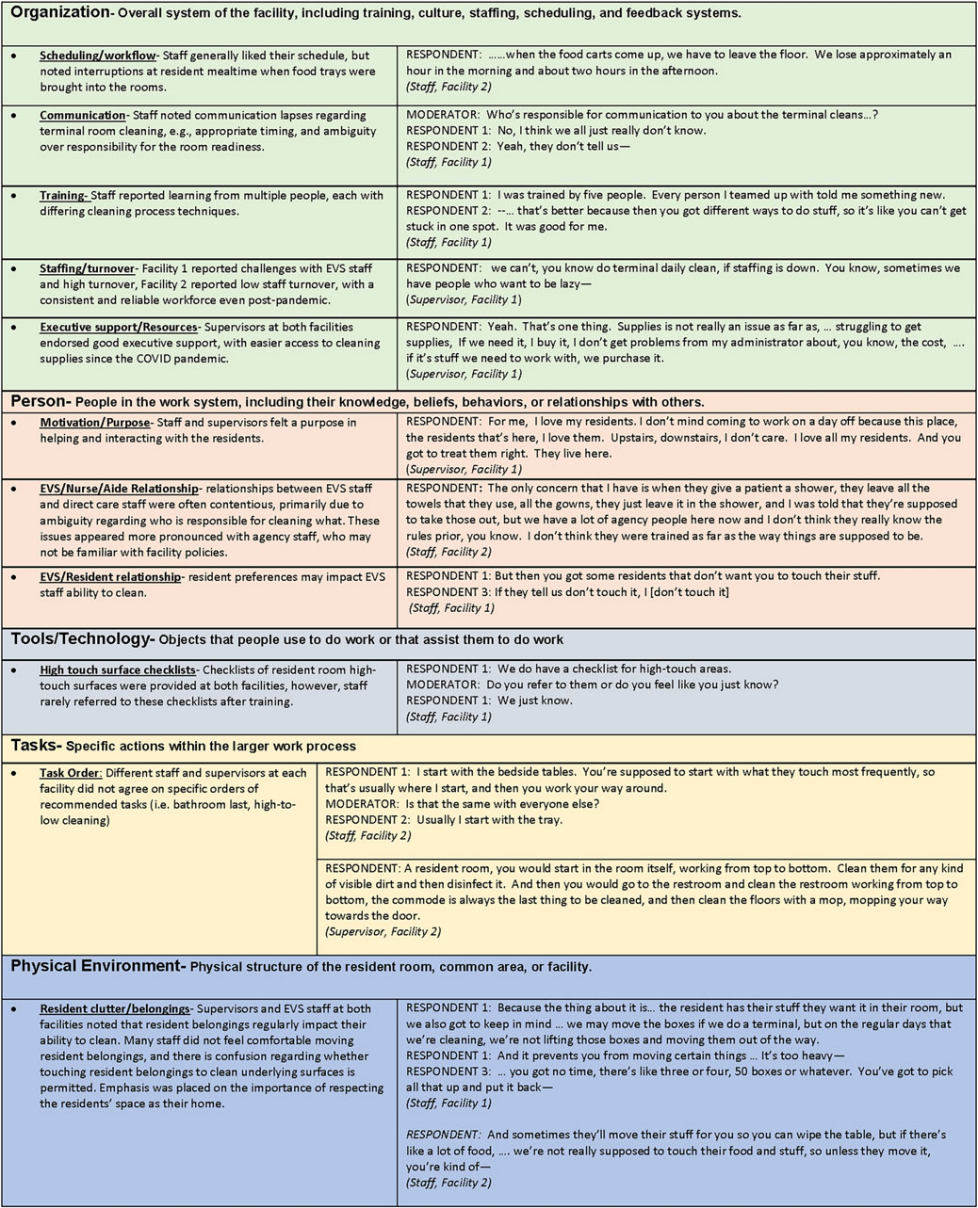
Focus group themes and quotations based on the 5 components of the SEIPS model.

Core themes and sub-categories of focus groups are depicted in Figure 3.

## Discussion

In this transdisciplinary mixed-methods study, we used environmental sampling and correlated results with direct observations and focus groups to better understand MDRO bacterial burden across LTC and its implication for improving environmental cleaning practices. Our findings revealed high levels of MDRO contamination across all sampled areas, with resident rooms demonstrating the greatest bacterial burden–particularly on surfaces closest to the resident. Direct observations of cleaning processes detected major deviations from basic infection prevention policies in over 50% of observations, suggesting there is a significant gap between recommended policies and actual practices among EVS staff. Focus groups identified key system-level barriers including lack of clear delegation of roles, inadequate communication and collaboration among different healthcare roles, frequent interruptions by residents and other healthcare workers, and inconsistent training practices. These barriers likely impact the efficiency and effectiveness of EVS work and are reflected in the high MDRO environmental contamination across the facilities.

### High percentage of MDROs recovered from all areas

MDRO environmental contamination is pervasive in LTC settings, and our findings are consistent with previous reports.^9–10^ While prior studies emphasized the need for improvements in environmental cleaning practices in LTC, this analysis, performed after the COVID-19 pandemic, when environmental cleaning was strongly emphasized, suggests opportunities for improvement remain.

While previous studies have found higher MDRO contamination in common areas,^10^ our data showed higher MDRO positivity in resident rooms compared to common areas. It is possible that the COVID-19 pandemic may have impacted these findings. In both facilities, an additional EVS staff member was assigned a “flex” position, responsible for focusing solely on additional disinfection of common areas, hallway railings, and elevators; this intervention was implemented in response to the emphasis on environmental cleaning in the early days of the COVID-19 pandemic.

Facility 1 had a higher overall MDRO burden than Facility 2; this was likely a reflection of the acuity of the residents in each facility–Facility 1, located in an urban setting, had a higher percentage of patients undergoing hemodialysis and long-term ventilator care patients, who are more likely to harbor resistant organisms.

### Higher bacterial burden in areas closest to the resident bed

Collecting composite samples of HTSs in different zones of the resident room allowed us to use this data to inform practical methods for EVS workflow. For example, quantitative bacterial burden was highest in Composite 1, which included samples closest to the resident bed. This may be because residents spend most of their time in bed and are more likely to shed bacteria in the areas directly around them. Our focus groups and observations revealed that most of the time the residents were in the room during cleaning, and staff were often hesitant to move resident belongings due to resident preferences, which impacts their cleaning process.

A mixed-methods review across Veterans Affairs long-term and acute care facilities^2^ echoes these findings, which reported more effective cleaning when the patient was absent. Specific education regarding the importance of frequent and thorough disinfection of surfaces around the resident, including tray tables, bed rails, and call switches, should be incorporated into EVS staff training. Clear expectations and methods to organize resident belongings to ensure tray tables and HTSs close to the resident are cleaned may be an important area of focus. For example, providing shower caddies to organize resident belongings may help to facilitate more efficient ways to move and clean resident items.

### Bacterial burden higher on long-term care units than any other sampled units

Overall bacterial burden was higher in LTC units, where residents often remain in the room for years, compared with other units, including short-stay and ventilator units. Our focus groups with supervisors and staff revealed potential causes for this accumulated bacterial burden. “Terminal” cleans, which include more in-depth cleaning of the resident room and typically require the resident to leave the room, are scheduled to occur monthly even for residents in long-term units who are not moving out of the room. Supervisors reported difficulties in both scheduling and staffing terminal cleans.

Missing or skipping terminal cleans can lead to bacterial buildup over time, and long-term units may be particularly vulnerable to this, as resident belongings may impact the ability to clean under accumulated personal effects. Incorporating clear schedules of terminal cleans into daily staffing workflow for long-term units may help alleviate some of this confusion and reduce the risk of missed or rushed terminal cleans.

### MRSA most prevalent organism identified, and ventilator units more likely to have multiple MDROs

MRSA was the most prevalent organism on environmental surfaces, which is concurrent with previous studies of both resident colonization and environmental contamination in LTC.^11–13^ Resident rooms in ventilator units were more likely to be contaminated with 2+ MDROs. This is consistent with prior studies showing environmental contamination of the room is more likely when a resident is colonized with a resistant organism,^12^ because residents residing in ventilator units are more likely to be colonized with an MDRO due to specific risk factors (ie, higher healthcare exposure, indwelling devices).

Emphasis should be placed on the importance of frequent and thorough environmental cleaning of ventilator units and resident rooms with known colonization status, including delegation of cleaning of shared medical equipment.

### Frequent practice deviations from facility policy

Our direct observations of EVS workflow showed frequent deviations from basic environmental cleaning policies, including cleaning from high areas to low areas to prevent particles falling to the floor after cleaning the floor, and cleaning from clean to dirty areas to prevent further contamination. While supervisors in focus groups clearly emphasized these policies and reported they were incorporated in basic training, interviews with EVS staff did not reflect strong knowledge of these policies. Training of EVS staff in both facilities was provided largely by shadowing, which resulted in significant variation in practices across staff members. These deviations reflect the need for more frequent observation and directed feedback by supervisors to ensure all staff are aligned on appropriate practice and are corrected prior to training new staff members. Additionally, reminders on these basic practices could be posted throughout patient rooms so that sequence of cleaning can continue to be emphasized even when supervisors are not present to correct staff.

### Limitations

While we did record whether or not a resident was known to be colonized by the nursing home based on standard passive surveillance practices, the number of residents known to be colonized with an MDRO was too low to identify whether there was a significant correlation between the resident’s colonization status and MDROs found in the environment. Additionally, while our study employed a robust bacterial plating method, further assessment of the isolated bacteria through molecular speciation via direct sequencing would have improved our understanding of the range of resistant microorganisms affecting the facilities.

It is also possible that our observations underestimate deviations from policy and missed HTSs due to the Hawthorne effect, as EVS staff were aware of the observer throughout their cleaning process. Despite this, we still saw high deviations from facility policy and missed high-touch surfaces, reflecting a need to address identified barriers. While we performed this study at two facilities with different corporate structures in order to identify organization-based differences which may impact findings, additional larger studies should be implemented to augment generalizability.

## Future directions

This transdisciplinary approach to environmental cleaning clarified the significant burden of MDROs contaminating the environment in LTC and the need to address gaps in environmental cleaning practices. Leadership engagement in the importance of environmental cleaning for resident outcomes, paired with standardized training practices with regular oversight and feedback may help prevent major deviations from policy. Efficiency and effectiveness (observed cleaning of HTSs) was impacted by frequent interruptions and clutter due to resident belongings. Further, many EVS staff reported a lack of teamwork with other healthcare roles, leading to missed HTSs (eg, shared medical equipment) and communication errors. Future interventions may include implementation of fluorescent gel sampling^14,15^ in LTCs for feedback and training opportunities, daily huddles with CNAs and EVS to clarify terminal cleaning schedules and specify roles for cleaning, and education to residents and family members regarding the impact of excessive clutter and resident belongings on proper cleaning and disinfection.

## Supporting information

10.1017/ash.2026.10324.sm001 Katz et al. supplementary material 1 Katz et al. supplementary material

10.1017/ash.2026.10324.sm002 Katz et al. supplementary material 2 Katz et al. supplementary material

10.1017/ash.2026.10324.sm003 Katz et al. supplementary material 3 Katz et al. supplementary material

10.1017/ash.2026.10324.sm004 Katz et al. supplementary material 4 Katz et al. supplementary material

## Supplementary material

The supplementary material for this article can be found at https://doi.org/10.1017/ash.2026.10324.
