# Supplementary figures and images for: Environmental contamination and cleaning practices in long-term care: a transdisciplinary mixed-methods study
Source: Antimicrob Steward Healthc Epidemiol. 2026 Apr 7;6(1):e77. doi: 10.1017/ash.2026.10324 (PMC13104514; doi:10.1017/ash.2026.10324)

**Supplementary Figure 1**

**
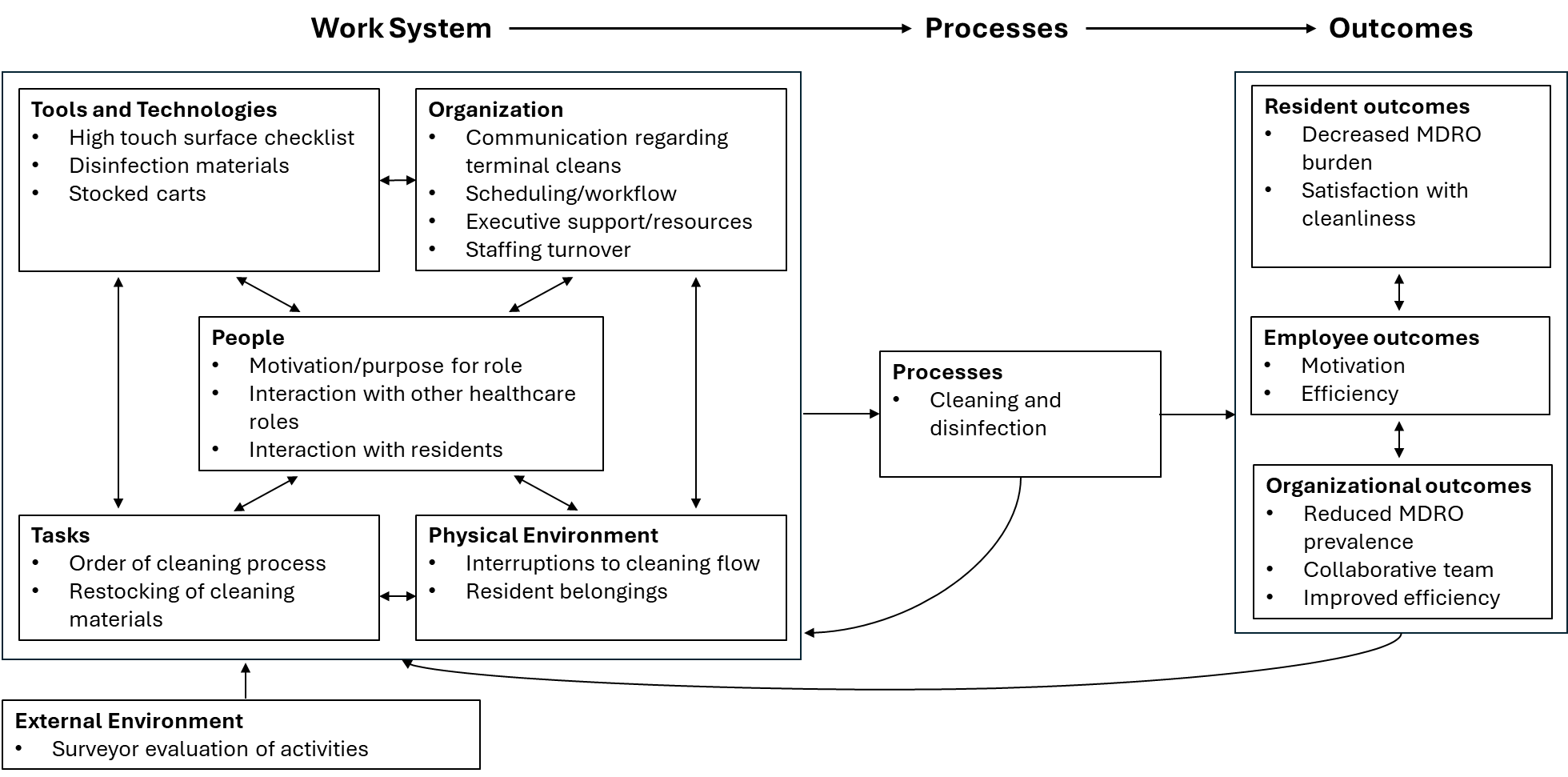
**

Supplement: Katz et al. supplementary material 1 — Katz et al. supplementary material [file S2732494X26103246sup001.docx]
