# Supplementary material for: Environmental contamination and cleaning practices in long-term care: a transdisciplinary mixed-methods study
Source: Antimicrob Steward Healthc Epidemiol. 2026 Apr 7;6(1):e77. doi: 10.1017/ash.2026.10324 (PMC13104514; doi:10.1017/ash.2026.10324)
Supplement: Katz et al. supplementary material 2 — Katz et al. supplementary material [file S2732494X26103246sup002.docx]

**Supplementary Table 1**

*Structural Assessments of Directors of Nursing, Maintenance, and Environmental Services at Facilities 1 and 2.*

| **Structural Assessment Questionnaire** | **Facility 1** | **Facility 2** |
| --- | --- | --- |
| **Key Points** | | |
| Number of Certified Beds | 150-200 | 50-100 |
| Average Residents/Day | 150-200 | 50-100 |
| Nurse Turnover Rate | 56% | 44% |
| Nurse hours/resident/day | 4.35 | 4.41 |
| 1. **Director of Nursing Assessment** | Conducted October 21, 2021 | Conducted November 30, 2021 |
| ***Roles*** | | |
| A1. Please state your race, ethnicity, and gender. | Caucasian Female | Caucasian and male |
| A2. How long have you been in your current role? | Six months | 2.4 years |
| A4. What was your work experience prior to your current role? | Assistant Director of Nursing and Performance Improvement Nurse | ADON, clinical coordinator, shift supervisor |
| A5. Have you received any specific infection control training? | No | Yes, Beacon Institute |
| ***Organization*** | | |
| A6. Do you have a designated infection preventionist (IP) or IP team in the facility? | Yes | Yes |
| A7. Is the IP a full-time role for this individual? | Yes | No |
| A8. If No, please indicate which of these activities also are performed by that individual: administrator (DON), quality manager, staff education/development, employee health, direct resident care, other. | *N/A* | Quality manager, staff education |
| A9. Is there a team or committee in your facility that reviews infection control-related activities (e.g., reports, policies, and procedures)? | Yeah. We review everything once a month at QAPI, and then if there’s any concern, so we do a morning, every morning a clinical meeting, so if there’s any infection control concerns from the day prior we bring it up there, and then also a weekly risk meeting. | We have stand-up and stand-down meetings every day, so we talk about it every day. |
| A10. If YES, how frequently does this committee meet? Annually, quarterly, monthly, weekly, other? | Monthly but then we do a weekly risk meeting [where] we also review infection control. As far as like statistics and all that, that’s just every month. | QAPI meets monthly. |
| A11. If YES, is environmental cleaning or facilities management a member of this committee? | Yes | Yes |
| A12. How much do you feel your facility stresses the importance of infection prevention on a scale of one to five, with five being the most? | Five | Five |
| A13. What is your ratio of RNs/residents? CNAs/residents? RTs/residents? OPN/residents? (per shift) | Per day shift:  1:8 GNA, 1:8.5 RN | Day shift:  1 GNA: 9 residents, RN: 1:11  Night shift:  1 GNA: 15 residents. |
| A14. What are your policies on visiting hours? | We don’t have any visiting hours. We have like suggested hours because we don’t want them to come in and have a party in the middle of the night, but they’re allowed to come in whenever they want as far as time goes. And right now we are not in outbreak status so there’s no restrictions on visitation right now. | We really can’t restrict visiting hours with the regs, so we try to have all visitors in and out by 9 pm. |
| A15. What are your protocols for placing new residents on precautions? Who is responsible for this? | if they are fully vaccinated, so 14 days past their second dose or their one dose of J&J, then there’s no isolation for that patient.  If they come in and they’re not fully vaccinated and past their 14 days, then they go on observation and by themselves in a private room for 14 days. | Really anybody can do it, if it’s a new admission from the hospital or a newly diagnosed infection or something like that, I mean, the nurse assigned to the patient would be the primary person to initiate it. But our infection control nurse, and she makes rounds through the building, you know, all throughout her shift while she’s here, and usually they would bring anything questionable to my attention as well. |
| A16. What is your facility’s capacity? |  |  |
| A16a. Total number of facility beds: | 150-200 | 50-100 |
| A16b. Number that are short stay: | The entire facility is dual certified-we have short-term stays sprinkled throughout the building into the long-term care units. | 20% short stay |
| A16c. Number of ventilator care beds: | 50 | 0 |
| A16d. Total number of resident rooms | 126 | 35 |
| A16e. Number that are SINGLE rooms: | 48 | 22 |
| A16f. Average daily census: | 150-200 | ~50 |
| A16g. Average proportion of short stay residents, in the past year: | 40 | 16 to 19 |
| A17. Which of the following primary service types are provided in your facility? (Yes/No/Don’t know) |  |  |
| A17a. Long-term general nursing care? | Yes | Yes |
| A17b. Long-term memory-need care? | No locked unit | No locked unit |
| A17c. Skilled nursing/short-term (subacute) rehabilitation? | Yes | Yes |
| A17d. Long-term psychiatric (non-dementia)? | Yes | No |
| A17e. Ventilator? | Yes | No |
| A17f. Bariatric? | Yes | Yes |
| A17g. Hospice/Palliative? | Yes | Yes |
| A17h. Other primary services? Please specify. | Wound care | *n/a* |
| A18. Which of the following clinical services are available in your facility (Yes/No/Don't know): |  |  |
| A18a. IV infusions using central lines? | Yes | Yes |
| A18b. Hemodialysis? | Yes | No |
| A18c. Management of residents with a tracheostomy? | Yes | Yes |
| A18d. Dedicated facility or contractual staff to provide wound care? | Yes | Yes |
| A18e. Dedicated facility or contractual staff to perform blood draws? | Yes, nurse or phlebotomy staff | Yes |
| A18f. 24-hour a day on-site supervision by an RN? | Yes | Yes |
| ***Training*** | | |
| A19. How are staff trained on infection prevention and device care protocols? Mentor, in-service? | Upon hire during orientation  Monthly IP refreshers | Upon hire during orientation  6 week refreshers/inservices |
| A20. How are new policies introduced? | Corporate nurse executive sends mass email, goes to IP and nurse managers then distributed via in services | In-services, text message, email |
| A21. Is there any follow up/refresher training? Describe it? | No. There’s like, it depends, so if something’s identified, then yes, but there’s not like a schedule for each policy.  Skilled one-on-one checks by IP on the floor | Handwashing surveillance and PPE refreshed every 6 months |
| A22. Duration and frequency of training? Yearly? Mandatory/voluntary? | On hire | On hire, then probably twice a year |
| ***Environment*** | | |
| A23. How are your handwashing stations set up? | We have sinks in the residents’ rooms in the bathroom, then there’s sinks in the clean utility, soiled utility, the med room, and staff bathroom and then there’s shower rooms that also have sinks. And then also the nurse has a sink./ refillable soap dispensers | Upstairs there’s two sinks right at the nurses’ station, and there are one, two, two bathrooms in the hall and we have a café area as well. We’ve had that closed since COVID. So there’s four sinks readily available, and then there’s some other sinks, like there’s one in the clean utility room, the soil utility room, and the telemedicine room, where we have a very small in-house lab.  In resident rooms, there is dispenser soap and towels. |
| A24. How are your hand sanitizers set up? | We have hand sanitizer in the rooms on the wall, and then hand sanitizers on the walls in the hallways. | We have on-the-wall dispensers, typically spaced like every other room or so in the hallway. There’s going to be one at the doorway of every single patient room, and then all throughout common areas of the building.  And then we also got like the disposable pump kind and they’re everywhere, I mean, everywhere you can imagine. |
| A25. Are there any restrictions on the materials kept in the room (e.g., chairs, resident items)? | It depends, so as far as you can’t like have meds in there, if that’s what you mean. Like you can’t, any medicate-, like treatments cannot be in there. We encourage them not to stock like the linen and stuff, like basically hoarding supplies. | Mostly food kind of stuff. |
| A26. Where are disinfectants for clinical team members located (e.g., sani-cloth wipes)? | They’re all over the place right now. So when they stock it, they put some in the clean utility, but when you go on to the units you’ll see them on the nurses’ station. There’s also some that are kept on the walls in the hallways. Different equipments, like the weight coordinator, she usually keeps hers attached to, I think it’s her Hoyer lift. | They’re everywhere. They’re going to be pretty much on every common area you’re going to see multiple bottles. We have two different types as well, and we also write on the top of the lid the kill time with a black Sharpie so it’s very obvious. So all throughout common areas, on every single piece of rolling equipment there’s wipe attached to that. |
| A27. How are cleaning responsibilities delineated (EVS vs clinical teams)? | Actually I’m not sure. | In our facility we have nursing just responsible for cleaning any type of equipment that they use to provide patient care, and there’s going to be wipes on the piece of equipment, as well as a little tag saying, you know, wash in between, sanitize in between patient use. And then EVS is going to be responsible for everything else, like all the high-touch surfaces and that kind of stuff. |
| ***Tools and Technology*** | | |
| A28. EMR system? Paper based? And how does it work? And how is the system accessed? | PointClickCare (PCC). Computer-based unless there is an outage. We have a backup system that would be paper at that point. We have a generator so it’s usually not a problem. [PCC is accessed at] mobile carts and then at the nurses’ station, and then management teams have laptops. | PointClickCare (PCC), paper based physician orders and hard chart for each patient as well |
| A28a. What technology do you use for documentation (e.g., iPads, phones, computers, workstations on wheels)? | So we only use the, once I said, the laptops on the mobile cart and stuff, but we do use other devices in other ways, so we use telemedicine. | We have desktop computers, laptop computers, iPads, and then we have a couple pieces of equipment that has some type of a tablet device on it. |
| A28b. When do you document (e.g., after each care activity, at the end of your shift, throughout the shift)? | They’re supposed to do it after the care is provided. The GNAs tend to, it’s a mix. So some of them do some towards the end. Some do, like they pick up all the trays, so then they document the meals for everyone. Like they’re not going to stop in between each tray they pick up to do it, but they’ll chart for the entire assignment after they pick up the trays. The nurses should be documenting as soon as they administer meds or provide treatment for each individual patient. Oh, and then as far as the charting system, the GNAs, they have kind of like a small TV, like a monitor that’s on the wall in the hallways. That doesn’t go in the patients’ rooms, so GNAs don’t document in the patients’ rooms at all, but they do share those screens in the hallways. | They’re supposed to document at the end of each individual care item. That’s the expectation. |
| A28c. When and how is mobile technology cleaned? | So they should be wiping them down in between going in the patients’ rooms, and in between the shifts, so when they are leaving for the day they should wipe theirs down | Whoever is using it is responsible for cleaning it. |
| 1. **Environmental Services / Facilities Management Assessment** | Conducted October 21, 2021 | Conducted November 30, 2021 |
| ***Roles*** | | |
| B1. Please state your race, ethnicity, and gender. | Black, Caribbean, Male | Black, Female |
| B2. What is your current role? | Director of Environmental Services | Director of Environmental Services |
| B3. How long have you been in your current role? | Eleven years at current facility | Two months |
| B4. What was your work experience prior to your current role? | Thirteen additional years of experience in this role | Business office manager |
| B5. Have you received any training in this role? | Trained as a maintenance assistant | Yes- online (Spartan chemical) – clainjack training program, 16 modules and through service with Bill |
| B6. How many facilities do you manage (i.e., just this one, or others)? | Just this one | One |
| B7. Is your EVS/Facilities management with a contracted service or are you employed by the facility? | Employed by the facility | Employed by the facility |
| B8. Is this a full-time role for this individual? | Yes | Yes |
| B8a. If No, please indicate which of these activities also are performed by that individual. | *n/a* | *n/a* |
| ***Organization*** | | |
| B9. Describe the organization of the team (e.g., how many EVS staff). | Team members include housekeepers and floor techs, totaling about 30 staff members | Nine EVS staff report to them |
| B10. How much do you feel your facility stresses the importance of infection prevention and environmental cleaning? (scale of 1-5) | Four or five | Five |
| B11. How many rooms are your staff responsible for cleaning on average in a day? | 200 | 10 to 15 |
| B12. Describe the schedules for cleaning (i.e., one staff member per unit, when they clean common areas, etc.). | Two staff members are assigned per unit, where they split the rooms and common areas. Common areas include shower rooms, nursing stations, soiled utility rooms, clean utitlity rooms, rehab, and dining rooms. Throughout the day, a floor tech is responsible for trash, pulls linen, buffs rooms, and strips and waxes rooms. | Morning meeting 8 am,  Housekeeping 8-4, clean 10-15rooms/shift  Floor tech starts at 630 am cleans common areas and pulls trash |
| B13. Do you have specific policies in place for room turnover? | Yes | Yes |
| B14. Is there a checklist of which surfaces/items are cleaned by EVS and which by nursing/others? | Yes, but I’m not sure if nursing does | Yes, but focuses on common area high touch surfaces, no delegation |
| B15. What is staff turnover in EVC per 6 months? | Lost more than 50 percent of staff | None |
| B16. Is there daily cleaning of resident rooms? What length of time is allocated for EVS to clean those? | Yes, 20 minutes | Yes, 15-20 minutes or as needed |
| B17. Is there daily cleaning of shared spaces (e.g., gym)? What length of time is allocated for EVS to clean that space? | Yes, about 30 minutes | The high-touch areas are hit hourly, but it’s as needed, so after a meal or something. We also have, when staff come into a break room like this and use it, they’re required to wipe their surface off with a Clorox disinfecting wipe. We have signage posted throughout. |
| B18. Is the cleaning after a patient has left that room (e.g., discharge clean) different to a daily clean? How? | Yes, everything is bleached. When you’re doing the terminal, the only thing we’re really left out of that is the beds, so when they go in the room and the stuff on the windows they don’t have beds and stuff to do, so that’s why we get down 15 to 20 minutes, so they can produce that room as far as sweep , mop, wipe down, we wipe from the window, from the window over bed lights, over bed tables, dressers, the footboard on the beds because all our footboards have touch pads. | Yes. You have, this is a turnover, which is split, it’s for a full turnover, someone is discharged completely out of the building.  In this it’s also, it has a checklist for maintenance, so things that we check, and then this is a hospital turnover, so somebody went to the hospital, they’re likely coming back, so we have similarities but some differences, and mainly the maintenance side is the checklist on that. |
| ***Training*** | | |
| B19. How are staff trained on proper environmental cleaning? Mentor, in-service? | A mentoring process – after orientation, housekeepers train new staff, and then I watch the trainee do a room | We use the same training program that we have, the Clean-Check training. We’ll set up on a large screen here in the morning and do a video of in-services that people have not seen, and if it’s something that they have done and it’s just a refresher, and I can show you back in our office, I have binders of all those, what we’ve done, sign it, you know, in-services   And then we’ll have them work with a preceptor who’s more senior and have them spend time with them.  We’re big into training.  We won’t put somebody out on their own until we’re comfortable and they’re comfortable. |
| B20. How are new policies introduced? | Meetings | It’ll be through a morning meeting that we have, and then we have a sign-in sheet and we have a checklist. We don’t have the same staff here every single day, so [name] will make sure that we get all the staff covered, we don’t miss anybody. |
| B21. Is there any follow up/refresher training? If yes, describe it. | Yes, in-person training | Yes, usually during the morning meeting. If we have somebody that wasn’t here when we did a training yesterday but they’re here the next day, we’ll get them one to one and do the training with them, which takes 15 minutes or so. |
| B22. Duration of training? Yearly? Mandatory/voluntary? | Yearly | Throughout the year: we do a QA monthly. |
| B23. Is there environmental quality assurance or monitoring program in place? | No | We want to make sure that we have some good things on there that we’ve been training people on. Part of our QA is making sure this is covered. |
| ***Environment*** | | |
| B24. Where are cleaning materials stored? | In the central supply, on the lower level. | We have a housekeeping storage room back in the service hall. |
| B25. Is there different cleaning disinfectants for different rooms e.g. contact precautions? | No. | Yes. We have different chemicals that we use for different applications. [Name] probably has a checklist. We have the one that just says, here you go. This is something that we review with the staff regularly as well. |
| B26. How do EVS team members become aware of a resident on precautions? | It’s posted outside the door. | Normally our quality assurance will send out an e-mail. We have a stop sign on the door that tells you to use precaution, you need to gown up, things like that. |
| ***Tools and Technology*** | | |
| B27. What is your EMR system? Paper based? And how does it work? | Mainly paper. | Paper-based. |
| B27a. What technology do you use for documentation (e.g., iPad, computer)? | *n/a* | All paper. |
| B27b. Is there any required documentation for cleaning practices? If yes, where and how is it documented? | Daily cleaning sheets are documented on paper. | Yes, we use a bed card.  admissions gives us and I hand out to our staff and they check off what rooms they have done. |
| B28. Are there different cleaning products for different surfaces? How do EVS know which product to use? | No. The cleaning products to use are posted for the EVS. | (?) |
| B29. Describe your air handling system (i.e., when it was last updated, management of air flow in particular units). | It was posted on the carts. I now notice that some are missing, but it has the chemicals they use on the cart and their kill times. No sense of when it was updated. | (?) |
| **C. Maintenance Director Assessment** | Conducted March 16, 2022 | Conducted November 30, 2021 |
| ***Roles*** | | |
| C1. Please state your race, ethnicity, and gender. | Afro-Trinidadian. Male. | White. (Possible) German descent. Male. |
| C2. What is your current role? | Maintenance director: maintain the facility in good repair and conduct inspections and drills for safety, as it may relate to state requirements. | Maintenance for facilities. |
| C3. How long have you been in your current role? | Sixteen years. | Four years. |
| C4. What building systems are you responsible for maintaining? | I ensure we have monthly inspections and testing to maintain water for laundry, kitchen and domestic, and resident areas. In addition, I maintain building structure, HVAC, air movement, exhaust, maintaining heat, heat in the winter, and coolness in the summer. | Anything in the building. |
| C5. How many people are on your team to maintain the building facilities? | One. | I have one maintenance person and 11 housekeepers. |
| C6. Has your role at this facility changed over the years? | Lost support from personnel. | I became facility director about a year and a half ago. Before I was, and my badge still says the same, maintenance and EVS director, and we took on an EVS director, which we had one before [name] who got promoted to another facility, and I [13:15]. |
| ***Organization*** | | |
| C7. What does a typical day at work look like? | I start here average 7:30 and then generally I would leave around 5:00, 5:30, and typically workday, the first thing I do, make rounds every morning.  I have a routine.  I walk the same hall every morning, same floor, around the same time, you know, make rounds.  We have maintenance log books.  I would go through the books just to satisfy the residents’ needs first.  Having done that, I would always have some burning issue, some project, so resident needs on a day to day and projects.   Assign inspectors in the building doing electrical integrity. I would have some sprinkler inspection, fire alarm inspection, you know, kitchen-related stuff to be inspected.  It’s all a lot of, it is about 20, almost 20 different things that has to be done annually. | There’s times when things might happen, multiple things happen all at the same time, and it’s just the way it is. I start normally anywhere from 7:00 to 7:30. My actual time is 8:00, but I come in early just because I like to get ahead of things. I usually leave anywhere from 4:00 to 5:00. |
| C8. Describe your air handling system. | HVAC- I don’t know about the update, but they’re in good working condition.  We have four units on the rooftop.  [7:57] can’t off the top tell you. But we have those.  And we also have units on the lower level, which mean the ground.  That satisfy what we have here, what we call PTAC units, that’s individual units per room, so these room units are all individual to each room.  The halls and common areas are satisfied by the rooftop units. | We have a total of 22 split units, which are gas-fired furnaces. And they supply heating and/or air conditioning throughout the common areas. We have four ERVs, which are energy recovery units or also referred to as exhaust recovery units as well, and they’re air handlers as well. They’re maintained by a company called All Quality, which will come out quarterly and do inspections. We replace filters monthly as a facility. My maintenance person and/or myself will replace them monthly. We’ve upgraded the filters to MERV-13 at a significant cost. You’re very familiar with that, if you, if you can buy them, you buy them, you know.  Since COVID, right. We have, in addition to the 23 split units, we have two mini splits, which are for an elevator room and a communication room, which is just to make sure that they’re kept cool. We have a total of 50 ETAC units, which are a small shelf containing air conditioning and heating unit in resident rooms. They’re maintained by us regularly. We clean the filters. Every six months we’ll do a preventive maintenance on the unit itself, pull it out and clean it. |
| C9. When was it last updated? | Unsure. | 2013 |
| C10. Do you have an air management plan? | We have a preventative maintenance system that is developed by direct supply, which tells us when things are due. We adhere to that. | The system is controlled through an automation system, called Cray Control, and that gives me the ability to go onto a site, an app or a program, and I can change the temperatures in different sections of the building. |
| C11. What systems are currently in place to clean the air? | We have air scrubbers and HEPA filters. | Air scrubbers (through All Quality). |
| C12. Do you manage air flow in particular units? | And we also have units on the lower level, which mean the ground.  That satisfy what we have here, what we call PTAC units, that’s individual units per room, so these room units are all individual to each room. | The Cray Control system changes temperature of different sections of the building. |
| C13. What metrics do you use to evaluate the quality of the air? | Unknown. | No. |
| C14. What percentage of outdoor air is incorporated into your HVAC system? | Unknown. | We meet the minimum requirements. |
| C15. Are HEPA filters used? If yes, what MERV rating are they? | Yes, eleven to thirteen. | No. |
| C16. Are portable air filters used in the building? Under what conditions are they used? | Yes, we have air scrubbers that we use especially when we were in breakout status and stuff when we had COVID units and [11:03], but we do have air scrubbers… If we have a positive case, where we have isolation situation, we install in that room the air scrubber, which provides a negative pressure, so everything that is scrubbed goes out the window. | No. |
| C17. What parameters are monitored to ensure the HVAC is operating properly? | Regular inspections. | HVAC is monitored quarterly. We call them if anything breaks. |
| C18. What are your cleaning protocols for the air handling systems and duct work? | Ductwork for the laundry, we do have that as a quarterly. We have a contractor that comes and cleans the laundry [13:22]. The others, we change filter. | We have a contract with All Quality. They replace filters for us on quarterly inspection. |
| C19. Describe your potable water system. |  |  |
| C19a. What is the source for the potable water (i.e. municipal water or private well)? | City water. | Municipal water (?) |
| C19b. Do you further treat the water on-site (filtration, softening, ion exchange etc.)? | Yes, we have a system that adds chlorine for monitors. | (?) |
| C19c. Do you have a water management plan for the building? If yes, is it written down? If not, please describe. | Chemstar, test for Legionella via the state | I have a building water management plan for helping prevention of Legionnaires, and I could show you that in more detail when we go back to the maintenance office.  We review that annually with the safety committee, have a flow chart and all that as to what water comes in and what it does and systems, things that we do to help prevent it.   We have daily all housekeepers and EVS, they run water in all drains, treat all drains with a biomatic, which is enzymes to help keep the drains clear.  We replace the water filters, which are for our ice makers every six months.  When we replace them, we date it, they’re tagged, and I manage this through a system called Express Maintenance.  Building water management.  Oh, daily we test temperatures throughout the building.  We check to make sure boilers are running properly, circulation valves are turning, and that’s done daily, or Monday through Friday that is done.  The maintenance person isn’t here on the weekend.   Yeah, you actually just reminded me, there’s another thing, and again, this will be all, if I missed anything, you’ll see it in this thing I have, but we also, monthly we do a drain and purge.  We call it a drain and purge.  We don’t completely drain it.  But we run the hot water tanks, which we have two of them in our boiler room.  We’ll run them into a drain for about 10, 15 minutes or more.  Another thing that we do which is also part of the same Legionnaires prevention is we clean all showerheads with a bleach solution, and that’s done by my maintenance person once a month. |
| C19d. Is the water in the building regularly monitored?  If yes, who does the sampling and analysis? | Yes, they are sent to a lab at least once a month. |  |
| C19e. Please describe the hot water system (central hot water tank, on-demand hot water etc). What temperature is the hot water set to? | It would supply our main hot water is supplied to deliver 116, you know, because by regulation we can’t do more than 120 for domestic, but for the laundry, we have individual system down there that boost the hot water and delivers 150 degrees, you know, and what goes to the kitchen would also be boosted at the dish machine, since they required to rinse at I think 180, 188 and to wash at 160. | 100-120 |
| C19f. Is the building water regularly flushed? What is the flushing regime? Who manages the flushing regime? | You don’t know.  Then they want to know what the flushing regime is, like how do they, how do you get the waste out of the, it just goes to the city.   What waste?  Oh.   I think it goes to the sewage, to the sewer.  We don’t have anything but sewage goes to the city treatment. | (?) |
| C20. Describe your waste management system. |  |  |
| C20a. Is the wastewater system connected to a municipal sewer or septic system? | Municipal. | Municipal |
| C20b. How are soiled materials stored and what is the disposal protocol? | Okay, so we have BSW.  I don’t know what the abbreviation is but that is a company that removes medical waste.  We have the laundry, of course, that deal with soiled linen and stuff.  We have a compactor system that removes other waste, yeah. | Trash? Okay.  The aides will clean and contain and they’re required and instructed to, and this is, again, a DON confirmation, and I remind them when I do a safety tour, they need to tie the bags, remove it from the room with the bag tied, and of course de-glove and all that, follow the right procedures, and get it to a soiled utility room.  Put it in a soiled utility room, where it will be covered in a large trash hopper.  Our floor tech will regularly pull trash out of these soiled rooms.  They have certain times, [name] would probably know better the times than I do, but there’s a morning, first thing in the morning is one of the things they do, they want to pull the trash out because we’ve got to be out of the halls before food carts get on the floor anyway.  That’s why the floor tech starts at 6:30 a.m.  And then they take it to a dumpster which we have out back.    Soiled laundry is the, is pretty much the same, because we put it in the bags--  Double bag, you know, double bag it--    --and then put it in the soiled utility room.  Then laundry does their pull.  If there’s anything on it, they’ll clean it in laundry using the proper PPE to pre-treat it or take something off of it if there’s feces or something on a garment. |
