# Supplementary material for: Environmental contamination and cleaning practices in long-term care: a transdisciplinary mixed-methods study
Source: Antimicrob Steward Healthc Epidemiol. 2026 Apr 7;6(1):e77. doi: 10.1017/ash.2026.10324 (PMC13104514; doi:10.1017/ash.2026.10324)
Supplement: Katz et al. supplementary material 3 — Katz et al. supplementary material [file S2732494X26103246sup003.docx]

**Supplementary Table 2**

*Template observation data entry forms.*

**
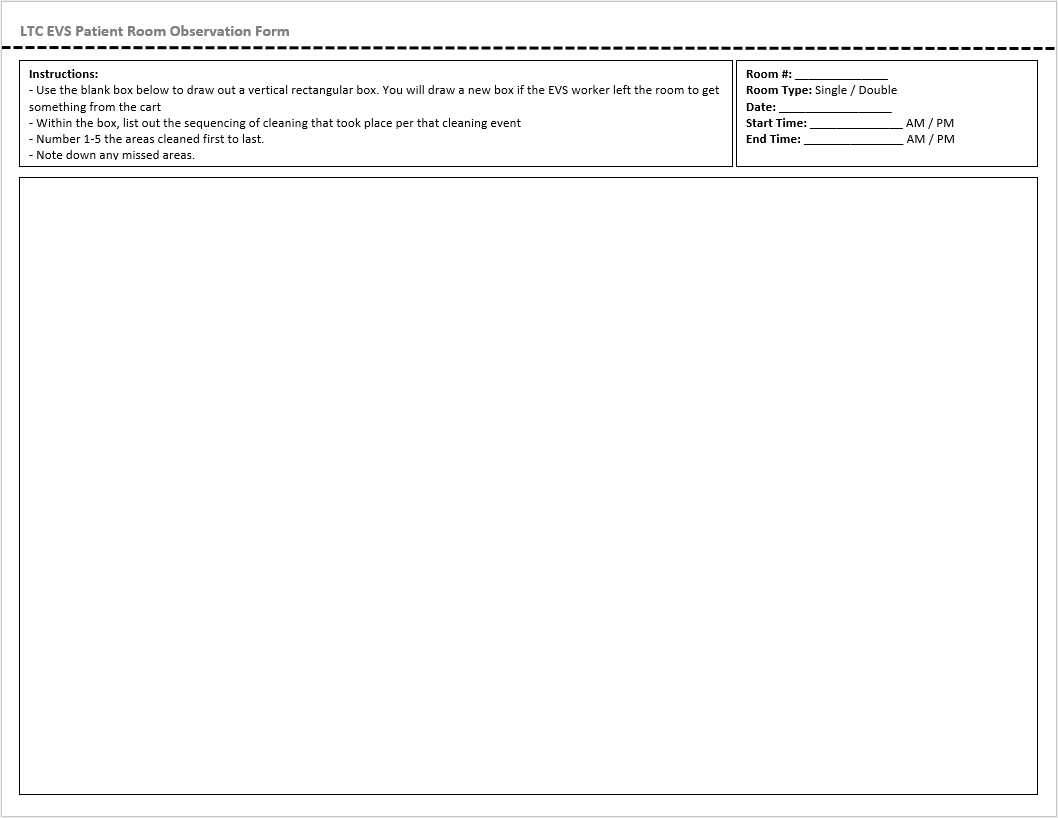
OBSERVER 01 DATA ENTRY FORM:**

**OBSERVER 02 DATA ENTRY FORM:**

1. Facility: FutureCare Irvington / Lorien Bulle Rock
2. Observer #1 name (diagram above): Morgan / Heather / Matiza / Ale / Clare / Taylor
3. Observer #2 name (respondent for questions): Morgan / Heather / Matiza / Ale / Clare / Taylor
4. Unit Type: LTC / Skilled / Vent
5. Room type: Single Patient Room / Double Patient Room / Gym / Dining Hall / Hallway
6. Room # (if applicable): ____________
7. Date (MM/DD/YYYY): ____________
8. Start time for EVS cleaning: ____________ AM / PM
9. Hands Barrier precautions (Is the EVS worker wearing gloves): Yes/No
10. Contact precautions: Yes / No
    1. If yes, was PPE worn: Yes / No
11. Hand hygiene prior to room entry: Yes / No
12. Cart placement: Inside / Outside
13. Resident(s) in room: Yes / No
    1. If yes, document location: Bed / Chair / Bathroom / Table (dining hall) / Equipment (gym)
    2. If yes, document number of residents in room: ____________
14. Visitors in room: Yes / No
    1. If yes, document number of visitors in room: ___________
15. Healthcare workers in room: Yes / No
    1. If yes, document number of HCWs in room: ___________
16. Document disinfectant(s) used:
    1. [Disinfectant 1]: hdq.C2 Cleaner Disinfectant/NABC Bathroom Cleaner/ Bio Renewables Glass Cleaner 18/ Damp Mop 8/ Contempo Spotting Solution/ TNT/ Fuzion/ Spar San Q/ Clorox Bleach Germicidal Wipes/ Clorox Hydrogen Peroxide Wipes/ Clorox Total 360/ Bio-Matic/ Phos-Clean Bowl and Tile Cleaner/ Other: ___________
       - 1. Contact time applied: _______________ seconds / minutes
         2. Spray on surface then wipe / Spray on cloth then wipe
         3. Disinfectant wipe / Microfiber cloth / Paper towel / Cotton rag
    2. [Disinfectant 2]: hdq.C2 Cleaner Disinfectant/NABC Bathroom Cleaner/ Bio Renewables Glass Cleaner 18/ Damp Mop 8/ Contempo Spotting Solution/ TNT/ Fuzion/ Spar San Q/ Clorox Bleach Germicidal Wipes/ Clorox Hydrogen Peroxide Wipes/ Clorox Total 360/ Bio-Matic/ Phos-Clean Bowl and Tile Cleaner/ Other: ___________
       - 1. Contact time applied: _______________ seconds / minutes
         2. Spray on surface then wipe / Spray on cloth then wipe
         3. Disinfectant wipe / Microfiber cloth / Paper towel / Cotton rag
    3. [Disinfectant 3]: hdq.C2 Cleaner Disinfectant/NABC Bathroom Cleaner/ Bio Renewables Glass Cleaner 18/ Damp Mop 8/ Contempo Spotting Solution/ TNT/ Fuzion/ Spar San Q/ Clorox Bleach Germicidal Wipes/ Clorox Hydrogen Peroxide Wipes/ Clorox Total 360/ Bio-Matic/ Phos-Clean Bowl and Tile Cleaner/ Other: ___________
       - 1. Contact time applied: _______________ seconds / minutes
         2. Spray on surface then wipe / Spray on cloth then wipe
         3. Disinfectant wipe / Microfiber cloth / Paper towel / Cotton rag
    4. Other(s):
17. Document surface types cleaned (check all that apply):
    1. Low-touch surfaces:
       - Window sill
       - A/C Unit
       - Other (please specify):
    2. High-touch surfaces:
       - Wheel chair
       - Mattress/bed controls
       - Left bed rail
       - Right bed rail
       - Bottom bed rail
       - Top bed rail
       - Bed tray top
       - Bed tray drawer (only cleaned during terminals)
       - Bedside tabletop / handles
       - Dresser cabinet top / handles
       - Floating cabinet top / handles
       - Overhead bed lamp
       - Overhead bed lamp switch
       - Telephone
       - Remote
       - Bedroom hand sanitizer dispenser
       - Bedroom light switch
       - Bedroom inside door handle / frame
       - Bedroom outside door handle / frame
       - Therapy equipment
       - Bathroom light switch
       - Bathroom rail
       - Bathroom sink
       - Bathroom sink faucet
       - Bathroom soap dispenser
       - Bathroom toilet paper dispenser
       - Bathroom paper towel dispenser
       - Bathroom sharps container
       - Bathroom mirror
       - Bathroom toilet
       - Bathroom toilet handle
       - Bathroom inside door handle / frame
       - Bathroom outside door handle / frame
       - Other (please specify):
18. Was the therapy equipment cleaned/disinfected after use? Yes / No / Not applicable
19. Document percentage of resident belonging coverage on horizontal surfaces:

*Note: If one observer, remember to circle surfaces with resident belongings on diagram.*

- 1. <25% (low) | 25-50% (average) | >50% (high)

1. Document percentage of medical equipment coverage on horizontal surfaces:

*Note: If one observer, remember to circle surfaces with resident belongings on diagram.*

- 1. <25% (low) | 25-50% (average) | >50% (high)

1. Document the number and type of interruptions:

*Note: Document if EVS worker has to explain their task to patient/visitor, or has to address/assist a colleague verbally, or has to leave room for any reason other than going to their cart for supplies.*

1. **Ask:** Type of room clean: Daily / Terminal
   1. If terminal, is patient leaving the room or is this part of a standard weekly requirement?

*Note: Morgan noted they do try and do a terminal clean x1/week even if the patient is not leaving that room.*

1. End time for EVS cleaning: ____________ AM / PM
2. Hand hygiene after room exit: Yes / No
3. Document any other questions and corresponding notes here:

****** See Contextual Inquires in Next Page ******

**CONTEXTUAL INQUIRIES:**

1. **Ask**: Any barriers to cleaning the room?
   1. Prompts (in case cleaner doesn’t know how to answer):
      1. Most common type of interruptions encountered
      2. Presence of resident belongings
      3. Were belongings moved to access items? Yes/No
         - 1. What percentage of personal belongings moved
      4. Too many items in room (e.g., furniture, medical equipment, etc.)
      5. Size/orientation of room layout
      6. Resident preferences in sequence of cleaning
      7. Personal physical obstacles such as needing a ladder to reach certain areas due
   2. Document any strategies observed to counteract barriers in the moment.
2. **Ask:** Do you try to coordinate cleaning when the patient is out of the room? Always / Sometimes / Never
   1. If yes, who do you coordinate with?
   2. Do you receive patients’ schedules ahead of time?
3. **Ask (conversational):** How long have you been working in LTC? And as an EVS worker?
