# Supplementary material for: Environmental contamination and cleaning practices in long-term care: a transdisciplinary mixed-methods study
Source: Antimicrob Steward Healthc Epidemiol. 2026 Apr 7;6(1):e77. doi: 10.1017/ash.2026.10324 (PMC13104514; doi:10.1017/ash.2026.10324)
Supplement: Katz et al. supplementary material 4 — Katz et al. supplementary material [file S2732494X26103246sup004.docx]

**Supplementary Table 3**

*Demographics of baseline focus group participants at Facilities 1 and 2.*

| **Baseline Focus Group Characteristics** | **Facility 1 (N=14)** | | **Facility 2 (N=7)** | |
| --- | --- | --- | --- | --- |
| **Age** |  |  |  |  |
| 18-37 | 7 | 50% | 1 | 14% |
| 38-57 | 6 | 43% | 2 | 29% |
| 58+ | 1 | 7% | 4 | 57% |
| **Gender** |  |  |  |  |
| Female | 10 | 71% | 4 | 57% |
| Male | 4 | 29% | 3 | 43% |
| **Ethnicity** |  |  |  |  |
| Non-Hispanic | 11 | 79% | 6 | 86% |
| Hispanic | 0 | 0% | 0 | 0% |
| Unknown or Not Reported | 3 | 21% | 1 | 14% |
| **Race** |  |  |  |  |
| American Indian/ Alaska Native | 0 | 0% | 0 | 0% |
| Asian | 0 | 0% | 0 | 0% |
| Native Hawaiian or Other Pacific Islander | 0 | 0% | 0 | 0% |
| Black or African American | 12 | 86% | 1 | 14% |
| White | 0 | 0% | 6 | 86% |
| More Than One Race | 0 | 0% | 0 | 0% |
| Unknown or Not Reported | 2 | 14% | 0 | 0% |
| **Education** |  |  |  |  |
| Less than a high school diploma | 1 | 7% | 0 | 0% |
| High school degree or equivalent | 13 | 93% | 5 | 71% |
| Bachelor’s degree | 0 | 0% | 0 | 0% |
| Other (some college) | 0 | 0% | 2 | 29% |
| **Role** |  |  |  |  |
| Environmental Care Cleaning Associate | 12 | 86% | 4 | 57% |
| Floor Tech | 2 | 14% | 1 | 14% |
| Respiratory Technician | 0 | 0% | 0 | 0% |
| Physical Therapist | 0 | 0% | 0 | 0% |
| Other (Facility Director / Maintenance Support) | 0 | 0% | 2 | 29% |
| **Years of service in their role** |  |  |  |  |
| Less than a year | 2 | 14% | 1 | 14% |
| More than a year, less than three years | 2 | 14% | 1 | 14% |
| More than three years, less than seven years | 6 | 43% | 3 | 43% |
| More than seven years | 4 | 29% | 2 | 29% |
| **Years of service in the facility** |  |  |  |  |
| Less than six months | 4 | 29% | 0 | 0% |
| More than six months, less than a year | 3 | 21% | 0 | 0% |
| More than a year, less than three years | 2 | 14% | 3 | 43% |
| More than three years, less than seven years | 3 | 21% | 3 | 43% |
| More than seven years | 2 | 14% | 1 | 14% |
